# Efficacy and safety of a novel naltrexone treatment for dry eye in type 1 diabetes

**DOI:** 10.1186/s12886-019-1044-y

**Published:** 2019-01-28

**Authors:** Patricia J. McLaughlin, Joseph W. Sassani, Michelle B. Titunick, Ian S. Zagon

**Affiliations:** 1 Department of Neural and Behavioral Science, The Pennsylvania State University College of Medicine, MC H109, 500 University Drive, Hershey, PA 17033-0850 USA; 2 Department of Ophthalmology, Milton S. Hershey Medical Center, Hershey, PA USA

**Keywords:** Dry eye disease, Tear production, Corneal surface sensitivity, Naltrexone

## Abstract

**Background:**

Dry eye disease (DED) is a prevalent complication of diabetes and presents as reduced tear production and/or increased corneal surface sensitivity often with secondary ocular surface changes. This study examined the safety and efficacy of a proprietary new eye drop formulation for topical treatment of DED.

**Methods:**

Type 1 diabetes (T1D) was established in male Sprague-Dawley rats to study the efficacy and safety of the investigational compound that contained 20 μg/ml of naltrexone (NTX). Tear production was measured by the Schirmer’s 1 test, and ocular surface sensitivity was measured using an aesthesiometer. Diabetic rats received twice daily applications of a single drop (~ 0.02 ml) of the proprietary formulation (NTX-001) or vehicle onto one eye. For comparison, some diabetic rats received eye drops containing NTX in sterile Vigamox®. Safety was monitored by assessment of ocular histopathology in naïve male rats and naïve male rabbits receiving twice daily treatment of two drops for 30 days.

**Results:**

Dry eye in T1D rats was reversed within hours of a single treatment of NTX-001, and over a period of 10 days NTX-001 restored corneal sensitivity and reversed dry eye relative to values measured in diabetic rats receiving vehicle. In comparison to NTX dissolved in Vigamox®, the proprietary NTX-001 was more effective at reversing dry eye. Safety studies in naïve rats and rabbits revealed no visible ocular pathology after 30 days of treatment.

**Conclusions:**

An investigational new eye drop containing 20 μg/ml NTX effectively reversed tear film deficits and restored corneal surface sensitivity in diabetic animals without causing toxic side effects.

## Background

Tear deficient dry eye disease (TDDE) or aqueous dry eye disease (DED) is defined as an abnormal level of tear production related to either pathophysiology and/or environmental factors. It is a multifactorial disorder characterized by changes in tear film volume and ocular discomfort, and may have an inflammatory component [1, 2]. If left untreated severe DED may cause pain, corneal ulceration, and potential loss of vision [1, 2]. Even milder forms of DED may produce chronic discomfort and blurred vision. The prevalence of DED ranges from 5% in the United States to 34% in Taiwan and Japan, with increased risk due to female gender, advanced age, and autoimmune disease (in particular type 1 or type 2 diabetes) [3–7].

In the U.S. there are approximately 29 million individuals diagnosed with diabetes and another 86 million considered pre-diabetic [4]. Nearly 50% of all diabetic individuals will experience chronic DED accompanied by changes in tear film osmolarity [3, 5–9]. Most of the treatments are over-the-counter (OTC) or prescribed lubricants (artificial tears, gels, ointments), and their success is dependent on frequent application. Many treatments provide only temporary relief and most do not target underlying disease pathobiology [10]. The common prescription treatments for TDDE with inflammatory etiology are Restasis® (Rx) with an active ingredient of cyclosporine, and Lifitegrast (Xiidra®), a lymphocyte-function-associated antigen-1 antagonist approved by the FDA in 2016 [11, 12]. Both treatments are topical medications that target dry eye resulting from inflammation. Restasis® has limited efficacy, and requires a lengthy period of application (e.g., 4 weeks) before being effective [12]. In contrast to the OTC artificial tears, gels, gel inserts, and ointments that provide temporary relief, steroid-loaded punctal plugs or surgical treatment through punctual occlusion may be offered to maintain basic levels of tear retention in cases of chronic dry eye syndrome [10]. Thus, there is an urgent medical need for new topical prescription treatments.

Preclinical studies have reported that the opioid growth factor (OGF) – OGF receptor (OGFr) regulatory pathway has a role in reversing or ameliorating several ocular-related complications of diabetes. Extensive investigations on corneal wound re-epithelialization in normal animals have documented the integral role of OGF in cellular homeostasis [13–15]. Additional studies on the blockade of OGF-OGFr interaction by NTX demonstrated accelerated corneal wound healing in diabetic mice and rats [16–18]. Preclinical reports have demonstrated that topical application of NTX dissolved in Vigamox® reverses dry eye and restores tear production and ocular sensitivity in type 1 and type 2 diabetic animal models within hours of a single eye drop [19, 20]. A small Phase 1 study with healthy human volunteers reported tolerability to escalating dosages (up to 5 × 10^− 5^ M) of topical NTX dissolved in Vigamox® [21].

In order to transition preclinical knowledge to the clinic, a new formulation was required that replaced the antibiotic-containing carrier Vigamox® and converted the NTX molar concentrations to a more conventional equivalent of the active ingredient. The formulation discussed in this manuscript involves an eye drop containing 20 μg/ml NTX. NTX is a general opioid antagonist that is FDA approved at 50 mg per day for systemic treatment of alcohol and opioid dependence in comparison to the 5 × 10^− 5^ M concentration, a 1000-fold less concentration tested in our tolerability study [22] or used in the present studies.

The GLP-formulation used in the present study is novel in that it will target the underlying pathophysiology of some diabetic complications - the dysregulated OGF-OGFr regulatory pathway and elevated enkephalins. Laboratory and clinical studies have documented elevated levels of plasma OGF, chemically termed [Met^5^]-enkephalin, in diabetic animals [23, 24] and humans [25, 26]. Elevations of this inhibitory peptide may contribute to the delayed cell replication and aberrant corneal surface sensitivity observed in diabetes. NTX at this dosage blocks the interaction of enkephalins as neurotransmitters or as an inhibitory growth factor. Sustained blockade of the interactions between OGF and OGFr results in accelerated cell replication, and thus enhances corneal repair, restores corneal surface sensitivity, and reverses dry eye in animal models of diabetes [16–20]. The bridging studies reported herein investigated the efficacy in T1D rats and safety in naïve rats and rabbits of the proprietary GLP-compliant formulation of NTX. No changes in subject animal behavior were noted by any observer providing the eye drops. Identification of a safe and effective formulation will enable future clinical trials for treatment of diabetic dry eye.

## Methodology

### NTX-001 efficacy study of type 1 diabetic dry eye

#### Animals and induction of type 1 diabetes (T1D)

Male Sprague-Dawley rats (~ 160 g body weight) were purchased from Charles River Labs (Wilmington, MA) and housed at the Penn State University College of Medicine. The Department of Comparative Medicine provides 24 h veterinary care, and water and food were available ad libitum. Male rats were chosen for this experiment in order to maintain consistency with previously published investigations on NTX treatment of diabetic complications [16, 17, 19], and to facilitate comparisons across studies. All animal procedures were approved by the Institutional Animal Care and Use Committee and conformed to the ARVO Guidelines for the Use of Animals in Biomedical Research.

Animals were rendered hyperglycemic by intraperitoneal injection of 40 mg/kg streptozotocin (pH 4.5) on two consecutive days. Blood glucose levels > 250 mg/dL indicated hyperglycemia and type 1 diabetes (T1D). Non-diabetic rats were considered normal controls and were injected with sterile saline. Animals were used 5 weeks after becoming hyperglycemic [16, 17]. Inclusion criteria for the T1D rats were (i) at least a 10% loss in body weight relative to non-diabetic male rats [17], (ii) blood glucose levels > 250 mg/dL, and (iii) baseline Schirmer values less than 70% of the mean Schirmer scores for normal rats.

#### Treatment

T1D rats were randomized to treatment groups and received twice daily (~ 0830 and ~ 1600) application to only the right eye of eye drops (~ 0.02 ml/eye drop) containing (i) 20 μg/ml NTX (NTX-001), (ii) buffer/vehicle, or (iii) 5 × 10^− 5^ M NTX dissolved in Vigamox®. Controls included: (i) the left untreated eye in T1D rats; (ii) right or left eyes of T1D rats receiving only the Vehicle; and (iii) right or left eyes of normal animals. The treatment continued daily for 10 days and was completed within 8 weeks of the first STZ injection.

#### Measurement of tear volume

Tear volume was measured with Schirmer strips (Alcon Laboratories, Inc., Ft. Worth TX). Based on the size of the rat cul-de-sac and the pool of tear fluid, standard Schirmer strips were precut to 1 mm × 17 mm long using sterile, single-edged razor blades, and measurements were made following published procedures [19]. Strips were inserted into the cul-de-sac for 1 min. The length of wetting on each strip was measured to the nearest half millimeter using fine rulers calibrated in millimeters. Schirmer 1 test scores for T1D rats treated with NTX-001, buffer, or Vigamox® were collected after one drop (2–4 h), two drops (~ 24 h following the initial drop and 15 h following the second drop), four drops (~ 72 h following the initial drop), and at 10 days. Additional measurements on rats treated with NTX-001 or buffer were made at 96 h and 7 days.

Following cessation of treatment, tear volumes were measured daily in a subset of rats to determine when the dry eye returned.

#### Measurement of corneal surface sensitivity

Corneal sensitivity was measured using a Cochet-Bonnet aesthesiometer (Boca Raton, FL) [27]. Four measurements were taken for each animal and averaged; the end point was a blink response. The values (g/mm^2^) were determined directly from the manufacturer’s conversion table. Corneal surface sensitivity was measured at baseline, 48 h, and 240 h (10 days) following treatment in T1D rats receiving buffer or NTX-001, and in Normal animals.

#### Intraocular pressure

At the termination of the study, intraocular pressure (IOP) was measured in treated groups of diabetic rats. Briefly, IOP was measured on unanesthetized rats using a tonopen (Tono-Pen XL Tonometer, Medtronic, Jacksonville, FL) [27]. The mean ocular pressure was obtained from 4 readings per eye.

#### Data analyses for efficacy studies

Data were collected in as much of a masked manner as possible. Data are presented as means ± SEM, and were statistically analyzed using two-tailed t-tests or analysis of variance (ANOVA) with subsequent comparisons made by Newman-Keuls tests; GraphPad Prism Version 6 was utilized.

### NTX-001 safety studies in Naïve rat and rabbit

The bridging efficacy study was performed first and, upon determination of efficacy, the safety studies were conducted.

#### Rat safety studies

Ten male (126–150 g) Sprague-Dawley rats were purchased from Charles River Laboratories and acclimated to the facility with ad libitum access to food and water. The treatment regimen was initiated 7 days later and consisted of each rat receiving 4 drops (0.02 ml per drop) daily of the proprietary NTX-001 formulation (20 μg/ml) with two drops administered to the right eye at 0830 and two drops applied at 1630 h. Twice daily treatment continued for 30 days at which time animals were humanely euthanized with Euthasol (> 150 mg/kg sodium pentobarbital), and eyes proptosed and fixed in Davidson’s Modified Fixative. Histotechnicians embedded the eyes (pupil-optic (PO) orientation) in paraffin, sectioned and stained with hematoxylin and eosin; slides were labeled in a masked manner for assessment by a veterinary ophthalmic pathologist [HindSight, LLC].

Each section was examined by light microscopy for adverse effects resulting from administration of test agents. Comparable anatomical structures for each section were examined to include: optic nerve, choroid, retinal pigment epithelium, neuroretina, ciliary body, lens, cornea, anterior chamber and posterior segment, as well as extraocular structures. Raw data were entered in an accompanying Excel file so that re-examination of individual structures at a later date was facilitated.

#### Rabbit safety studies

Ten male 2 kg New Zealand white rabbits were purchased from Robinson Services Inc. (RSI) and acclimated for 2 weeks prior to any investigation. Rabbits were housed individually with ad libitum access to food and water. Five rabbits received 4 drops daily of the NTX-001 formulation for 30 day; 2 drops (0.05 ml per drop) at 0830 and 2 drops at 1630 h in the right eye. Five rabbits received 2 drops twice daily of the vehicle into the right eye; no treatment was administered to the left eye of either group. After 30 days, rabbits were euthanized following the AVMA Guidelines for the Euthanasia of Animals and were sedated with 30–40 mg/kg (body weight) ketamine and 3–5 mg/kg xylazine injected intramuscularly followed by intravenous infusion into the ear vein of pentobarbital (> 150 mg/kg). Eyes were proptosed, and tissues fixed in Davidson’s Modified Fixative containing formalin. Veterinary histotechnicians embedded the tissues in paraffin, sectioned through the optic nerve, and stained with hematoxylin and eosin for pathological assessment by the veterinary ophthalmic pathologist [HindSight, LLC].

#### Data analyses for safety studies

Qualitative assessments of the rat and rabbit eyes were recorded by staff during the course of treating the animals. In addition, the veterinary pathologist prepared formal documentation of ocular tissues.

## Results

### Clinical characteristics of T1D rats

**Fig. 1:**
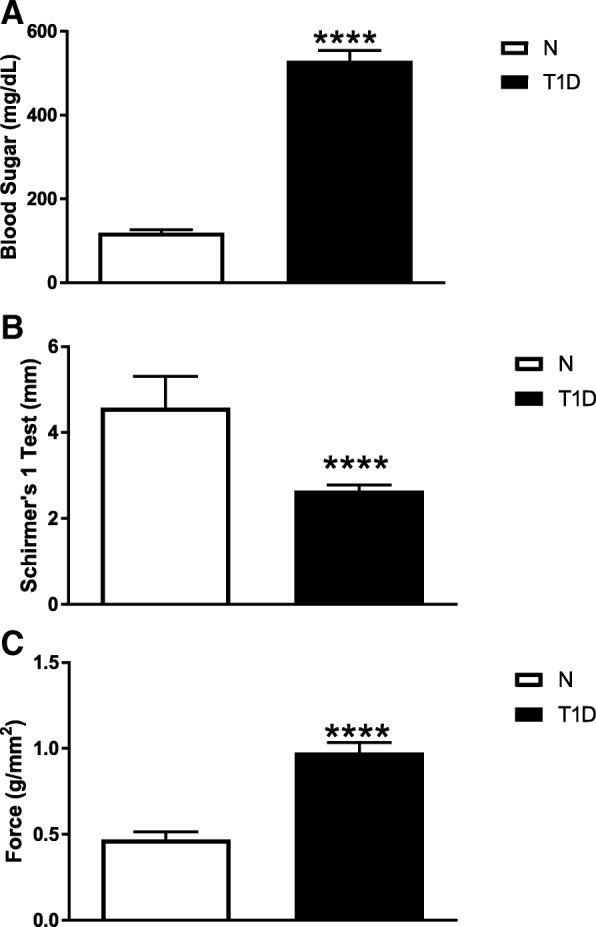
Baseline data of type 1 diabetic (T1D) rats included in the efficacy study of NTX-001 formulation. Histograms represent means ± SEM for (**a**) blood glucose (mg/dL), (**b**) Schirmer 1 test values (mm), and (**c**) corneal surface sensitivity as measured force (g/mm^2^) using a Cochet-Bonnet aesthesiometer. Data were analyzed using a two-tailed Student’s t-test. Values differed significantly between normal rats (N) and T1D animals at *p* < 0.0001 (****). Data reveal that T1D rats were hyperglycemic, had dry eye, and decreased corneal surface sensitivity

Hyperglycemia was confirmed by body weight and blood glucose levels. Mean body weight of rats at the time of STZ injection was 165 ± 2 g. At the time of initiating the study, normal rats weighed 386 ± 21 g in comparison to T1D rats that weighed 324 ± 7 g. Glucose measurements at the start of eye drop application were 530 ± 24 mg/dL for T1D rats and 119 ± 6 mg/dL for normal animals [Fig. 1a]. Baseline Schirmer tests revealed that right and left eyes of T1D rats were comparable with measurements of 2.5 ± 0.1 mm; right and left eyes of normal rats were comparable with measurements of 6.7 ± 1.3 mm [Fig. 1b]. Corneal surface sensitivity as measured by the Cochet-Bonnet aesthesiometer was 0.95 ± 0.06 g/mm^2^ for T1D rats and 0.47 ± 0.05 g/mm^2^ for normal rats [Fig. 1c]. All comparisons between baselines for T1D and normal rats were significantly different at *p* < 0.0001. Twenty-one T1D rats met the criteria of having dry eye and 10 were treated with NTX-001, 8 received Vigamox® + NTX, and 3 T1D rats received buffer only; 5 rats were non-diabetic and considered Normal (N). Criteria that excluded a number of rats in this study were insufficient or inconsistent dry eye values. Normal rats had low tear production during this study that was most likely related to low humidity levels in the animal facility during the experimental period of May through July; nonetheless, normal rats had tear volumes that were approximately 2.5 times greater than T1D values.

### Low tear production in T1D rats is restored by NTX-001

**Fig. 2:**
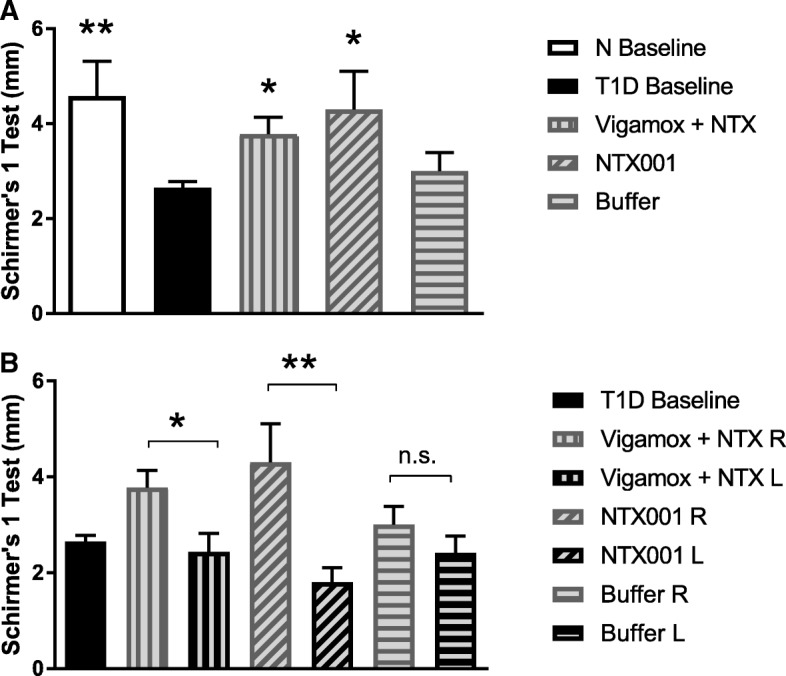
Reversal of dry eye in T1D rats following topical application of NTX-001 formulation. **a** Tear secretion measured 2–4 h following a single drop of new formulation (NTX-001), naltrexone dissolved in Vigamox® (Vigamox+NTX), or buffer. Values (mm) represent means ± SEM for Schirmer 1 test measurements in treated right eye. For comparison, baseline values of both normal (N) and T1D rats are included. Significantly different from T1D baseline at *p* < 0.05 (*) or *p* < 0.01 (**). **b** Schirmer 1 test measurements (mm) in the treated right eye (R) and untreated left eye (L) 2–4 h following a single drop of formulation (NTX-001), naltrexone dissolved in Vigamox® (Vigamox+NTX), or buffer. Values represent means ± SEM. Significantly different between right and left eyes at *p* < 0.05 (*) or *p* < 0.01 (**). n.s. = not significant. Data demonstrate that NTX-001 works quickly (within 2–4 h), restores tear secretion only in the treated eye, and is comparable in efficacy to Vigamox® + NTX treatment

**Fig. 3:**
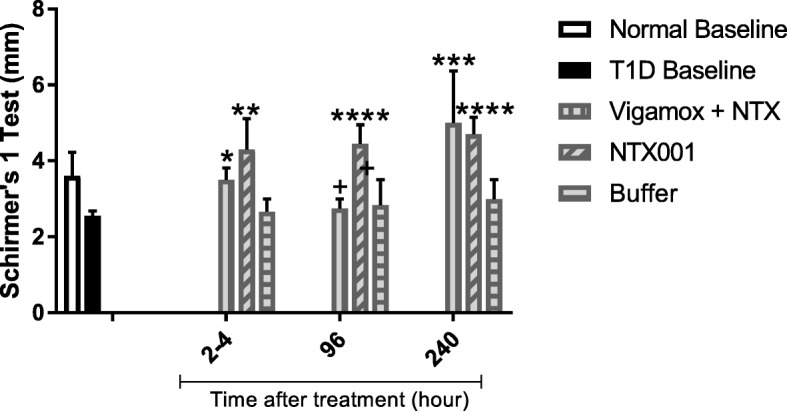
Efficacy of NTX-001 to restore tear secretion over a period of 10 days. Tear secretion (mm) was measured (2–4 h, 96 h, and 240 h) in the treated right eye of rats receiving Vigamox® + NTX, NTX-001 formulation or buffer; normal (N) baseline and T1D baseline values are included for comparison. Values represent means ± SEM; significantly different from T1D baseline values at *p* < 0.05 (*), *p* < 0.01 (**), *p* < 0.001 (***), and *p* < 0.0001 (****).Significant difference between Vigamox+NTX and NTX-001 was noted at 96 h (+, *p* < 0.05)

A single drop of NTX-001 within 4 h of treatment increased tear volume in the treated right eye of T1D rats receiving NTX-001 or Vigamox® in comparison to the baseline tear volume; one drop of buffer had no effect on tear volume [Fig. 2a]. Tear production in the treated right eye of T1D rats receiving NTX-001 or Vigamox® differed from that in the corresponding untreated eye [Fig. B]. Tear volume in both right and left eyes of rats receiving buffer was comparable. After 96 h of treatment (8 drops), tear volume was significantly greater in T1D rats receiving Vigamox®-NTX (3.6 ± 0.3 mm) or NTX-001 (4.3 ± 0.4 mm) relative to that recorded for T1D baseline (2.6 ± 0.1 mm); NTX-001 treatment also restored tear volume relative to buffer (2.7 ± 0.1) treatment [Fig. 3].

### Corneal sensitivity is restored following NTX treatment

**Fig. 4:**
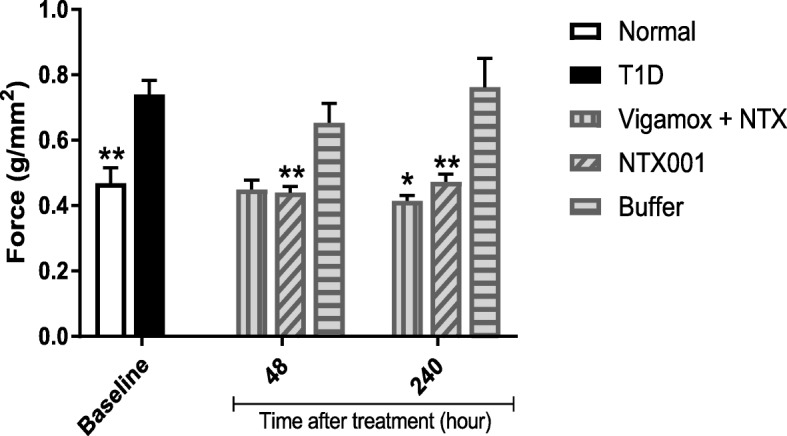
Corneal sensitivity as measured by force (g/mm^2^) required to elicit a blink response using the Cochet-Bonnet aesthesiometer. Diabetic rats (T1D) were treated in the right eye with Vigamox® + NTX, NTX-001 formulation, or buffer for 48 or 240 h. Baseline values for normal (N) rats and T1D rats are provided for comparison. Values represent means ± SEM. Significantly different from T1D baseline measurements at *p* < 0.05 (*) or *p* < 0.01 (**)

Baseline measurements of corneal sensitivity were measured by the Cochet-Bonnet aesthesiometer and revealed that the right and left eyes of T1D rats were comparable and required twice the force to elicit a response as required by normal rats. [Fig. 1c]. Corneal surface sensitivity was measured after 4 drops of NTX-001 (48 h post initial treatment) and after 10 days of treatment [Fig. 4]. After 4 drops of NTX-001, corneal sensitivity in the treated right eye of the T1D rats was restored to that of Normals, and thus the measurements differed significantly from those of T1D baseline and T1D rats receiving buffer (*p* < 0.0001) [Fig. 4]. At 10 days of treatment, the NTX-001 treated right eye continued to have comparable corneal surface sensitivity relative to non-diabetic, Normal rats, and thus required significantly reduced force measurements to elicit a response relative to baseline T1D values or to those in T1D rats receiving buffer.

### Intraocular pressure following NTX-001 treatment

IOP values as measured by tonopen (Tono-Pen XL Tonometer, Medtronic, Jacksonville, FL) in the unanesthetized NTX-001 treated eye (18.5 ± 1.9 mmHg) of T1D rats were comparable to those of Normal rats (17.2 ± 1.6 mmHg) after 10 days of treatment; IOP values in the untreated T1D eye were 10.9 ± 0.7 mmHg.

### Extinction time

After 10 days, topical administration of NTX-001 ended, and modified Schirmer 1 tests were conducted on a subset of rats that received daily NTX-001 in order to determine when the positive effect of restored tear production was extinguished. Using a score of 3.0 or less as indicating a return to “dry eye”, at 24 h after treatment cessation, only 1 of 8 rats had “dry eye”. At 48, 72, and 96 h, low tear volume indicating the return of dry eye was detected in 50, 20, and 75% of the remaining seven rats, respectively, receiving NTX-001.

### Rat safety studies

The veterinary ophthalmological pathologist received 20 masked slides of 2–3 sections/slide of treated (right) and untreated (left) eyes from normal rats receiving either NTX-001 (*n* = 5) or buffer (*n* = 5). At least one or more sections for every eye were of sufficient quality to be evaluated by the pathologist. Regarding rat pathology, no evidence of inflammatory, neoplastic or degenerative change was noted in any eye. Minor changes were reported in three eyes. Small amounts of amorphous eosinophilic debris were noted in the anterior chamber of the right eye of two rats and in the left eye of one rat, and a small retinal (outer nuclear layer) rosette was present in one eye. All were considered developmental lesions of no clinical consequence. Qualitative data on rats were based on observations by two graduate assistants who administered formulations twice daily to the normal and diabetic rats, as well as the veterinarians and animal handling technicians who were observing the animals at least once daily. No signs of distress immediately following application (e.g., squealing, crying, tearing, or vocalizations) were observed, and no redness or crusting in the treated eye was noted at the second application (7–8 h later) each day. At the end of the 30 day study, rat eyes appeared clear (no cataracts), with no excessive tearing.

### Safety study of rabbits

The veterinary ophthalmological pathologist received 20 masked slides of 2–3 sections/slide of treated (right) and untreated (left) eyes from normal rabbits receiving either NTX-001 (*n* = 5) or buffer (*n* = 5). Rabbit eyes were most commonly presented in sagittal orientation (cornea in normal anatomic orientation), and sections were variably distorted due to fragmentation of retina, cornea, or sclera. Overall, the optic nerve could not be adequately examined. The filtration angle was visible and in the normal anatomic orientation. The lens was present but sometimes fragmented in the sections. Individual cell types in various structures could be seen (including the retinal pigment epithelium), and nuclear and cytoplasmic detail was appropriate. The pathologic report indicated that there was no evidence of neoplastic or degenerative change noted in any eye. Minor or minimal inflammatory changes were noted in the conjunctiva of two eyes, both of which received no treatment. Qualitative assessments were made by the veterinary technicians who were treating the rabbits twice daily for 30 days. Over the course of one-month of treatment, no eye abnormalities were detected in rabbits, and there were no signs of redness, edema, irritation, or excess tearing or dryness. The rabbits displayed no signs of pain or distress upon administration of eye drops or post administration. At the end of the study, all rabbits were healthy and appeared to have normal vision.

## Discussion

Corneal complications in diabetic humans are manifested in several ways including decreased tear production, aberrant ocular surface sensitivity, and delayed corneal re-epithelialization [3–9]. These defects are also evident in animal models of diabetes, and each complication has its own underlying pathobiology. All of these complications have been reversed by topical application of NTX [16–20]. Our preclinical studies with diabetic mice, rats, and rabbits have utilized molar concentrations of NTX dissolved in Viagmox®, whereas in the present study we demonstrated that a proprietary eye drop formulation of NTX (20 μg/ml in a sodium carboxyl methyl cellulose carrier) was comparable or more effective at reversing dry eye in T1D rats than our original therapeutic of NTX dissolved in Vigamox® [19, 20]. The original dose of 10^− 5^ M NTX represents approximately 0.185 μg NTX per drop whereas the current study used a 20 μg/ml solution where 1 drop (~ 50 μl) provided 1 μg NTX which was significantly more than used in the earlier preclinical studies. The present efficacy studies spanned 10 days in length with follow-up occurring for several days after cessation of treatment; this time frame was longer than previously reported [19]. The safety studies were 30 days in length and resulted in testing an accumulated dose of 120 μg NTX. This direct total ocular application to naïve rats and rabbits of 120 drops was significantly more than that shown previously in our diabetic rat or mouse studies.

The T1D model of rats had significantly lower tear fluid levels and significantly less ocular sensitivity than normal rats. In summary, one eye drop of the proprietary formulation (NTX-001, 20/ml μg NTX) reversed dry eye (2–4 h measurements). Continual twice daily application resulted in significantly more tear production in eyes treated with NTX-001 relative to buffer at 96 h and 240 h. Following cessation of 20 applications of NTX-001, tear fluid levels comparable to those recorded in non-diabetic rats lasted up to 72 h. Furthermore, treatment of the right eye with NTX-001 reversed dry eye in that eye, and the untreated left eye was not affected suggesting that there was no crossover effect. Tear fluid levels were significantly greater in comparison to baseline T1D values, untreated eyes, and those receiving NTX-001 buffer or vehicle.

Comparison of NTX dissolved in Vigamox® to that of NTX-001 revealed that Vigamox® + NTX was effective at 2–4 h (one-drop), but less effective than NTX-001 at 96 h and 10 days. It is important to note that previous published reports used 10^− 5^ M NTX in Vigamox® applied 4 times daily, and the present study utilized 5 × 10^− 5^ M NTX dissolved in Vigamox® applied 2 times daily. Therefore, a direct comparison between the investigational formulation and NTX in Vigamox® is not possible. Finally, no gross visible pathology, irritation (rubbing), redness, or opacities were noted in naïve rats or rabbits receiving 30 days of treatment. The veterinary technicians did not observe any adverse behavioral responses in the subject animals after topical administration of the eye drop, suggesting that the eye drop did not cause even minor pain. At the end of 10 days, IOP values were within the range of IOP reported previously in diabetic and normal rats (24–26 mmHg) [27]. T1D rats had low tear volume several days after cessation of NTX treatment implying that this treatment does not cure dry eye, and that chronic treatment would be necessary for treatment of T1D associated dry eye.

The mechanisms associated with NTX-induced reversal of dry eye may be related to intrinsic ocular homeostasis. Studies on both type 1 and type 2 diabetes have reported elevated enkephalin levels, with specific levels of [Met^5^]-enkephalin (i.e., OGF), the inhibitory growth factor that reduces cell replication, shown to be elevated in both animal and human models [23–26]. Regarding the mechanism of reversing low tear production, the pathways most likely involved may be the peripheral sensory and motor nerves innervating the ocular surface [7]. With the knowledge that diabetics have elevated enkephalins that diminish responsiveness to stimuli at the sensory and motor levels, NTX may disrupt this endogenous opioid-receptor interaction and restore corneal sensitivity that in turn alters tear production. The observation that topical NTX restored corneal surface sensitivity should be examined further to determine whether this therapy may provide more than palliative care for ocular pain.

Blockade of OGFr by antagonists prepared as gels, ointments, or lubricants appear to be stable and tolerable alternatives for use as therapeutics [28]. In conclusion, we propose that the present data, along with our earlier preclinical studies, support the transition of this therapeutic modality into clinical trials for diabetic dry eye.

## Conclusions

This study reports that a novel GLP-compliant formulation of NTX used as a topical application is effective at reversing dry eye and restoring corneal surface sensitivity in diabetic rats. Gross and histopathologic study of the eyes from naïve rats and rabbits treated topically twice daily for 30 days with the formulation did not reveal any abnormalities. In summary, this novel formulation is effective and safe in preclinical studies, and data support the initiation of clinical trials for treatment of TDDE.

## Abbreviations

NTX: Naltrexone
OGF: Opioid growth factor
OGFr: Opioid growth factor receptor
STZ: Streptozotocin
T1D: Type 1 diabetes
